# Resistin facilitates VEGF-A-dependent angiogenesis by inhibiting miR-16-5p in human chondrosarcoma cells

**DOI:** 10.1038/s41419-018-1241-2

**Published:** 2019-01-10

**Authors:** Shiou-Sheng Chen, Chih-Hsin Tang, Meng-Ju Chie, Chun-Hao Tsai, Yi-Chin Fong, Yung-Chang Lu, Wei-Cheng Chen, Cheng-Ta Lai, Chuan-Yen Wei, Huai-Ching Tai, Wen-Yi Chou, Shih-Wei Wang

**Affiliations:** 1 Department of Urology, National Yang-Ming University School of Medicine, Taipei, Taiwan; 2 Division of Urology, Taipei City Hospital Heping Fuyou Branch, Taipei, Taiwan; 3 Commission for General Education, National United University, Miaoli, Taiwan; 4 Department of Pharmacology, School of Medicine, China Medical University, Taichung, Taiwan; 5 Chinese Medicine Research Center, China Medical University, Taichung, Taiwan; 6 Department of Biotechnology, College of Health Science, Asia University, Taichung, Taiwan; 7 Graduate Institute of Basic Medical Science, China Medical University, Taichung, Taiwan; 8 Department of Sports Medicine, College of Health Care, China Medical University, Taichung, Taiwan; 9 Department of Orthopedic Surgery, China Medical University Hospital, Taichung, Taiwan; 10 Department of Orthopedic Surgery, China Medical University Beigang Hospital, Yunlin, Taiwan; 11 Department of Medicine, Mackay Medical College, New Taipei City, Taiwan; 12 Department of Orthopaedics, MacKay Memorial Hospital, Taipei, Taiwan; 13 Division of Colon and Rectal Surgery, Department of Surgery, MacKay Memorial Hospital, Taipei, Taiwan; 14 Department of General Surgery, Taitung MacKay Memorial Hospital, Taitung, Taiwan; 15 School of Medicine, Fu-Jen Catholic University, New Taipei City, Taiwan; 16 Department of Urology, Fu-Jen Catholic University Hospital, New Taipei City, Taiwan; 17 Department of Orthopedic Surgery, Kaohsiung Chang Gung Memorial Hospital Medical Center, Kaohsiung, Taiwan; 18 Graduate Institute of Natural Products, College of Pharmacy, Kaohsiung Medical University, Kaohsiung, Taiwan

## Abstract

Resistin is an adipokine that is associated with obesity, inflammation, and various cancers. Chondrosarcomas are primary malignant bone tumors that have a poor prognosis. VEGF-A is a critical angiogenic factor that is known to promote angiogenesis and metastasis in chondrosarcoma. It is unknown as to whether resistin affects human chondrosarcoma angiogenesis. In this study, we show how resistin promotes VEGF-A expression and subsequently induces angiogenesis of endothelial progenitor cells (EPCs). Resistin treatment activated the phosphatidylinositol-3-kinase (PI3K) and Akt signaling pathways, while PI3K and Akt inhibitors or siRNA diminished resistin-induced VEGF-A expression. In vitro and in vivo studies revealed the downregulation of micro RNA (miR)-16-5p in resistin-induced VEGF-A expression and EPCs angiogenesis. We also found a positive correlation between resistin and VEGF-A expression, and a negative correlation between resistin and VEGF-A with miR-16-5p in chondrosarcoma patients. These findings reveal that resistin facilitates VEGF-A expression and angiogenesis through the inhibition of miR-16-5p expression via PI3K/Akt signaling cascades. Resistin may be a promising target in chondrosarcoma angiogenesis.

## Introduction

Chondrosarcomas are common primary malignant bone tumors that are difficult to diagnose and treat^1^. The most common age at diagnosis is between 30 and 60 years, with a peak appearing between ages 40 and 50 years and a male:female ratio of ~2:1^1,2^. Chondrosarcomas most frequently involve the scapula, sternum, ribs, and pelvic bones^3^ and their prognosis is poor, as they do not respond well to conventional treatments such as chemotherapy or radiotherapy^4^. Surgical resection is the cornerstone of treatment^5^. The lack of an effective adjuvant therapy for chondrosarcomas highlights the importance of developing novel treatments.

Mortality in cancer patients is mainly due to the metastatic spread of cancer cells to distant organs^6^. Increasing reports have concentrated on the effects of angiogenesis in cancer development and metastasis^7–9^. Tumor angiogenesis occurs as a result of the unbalance between pro- and anti-angiogenic factors^10^. Vascular endothelial growth factor-A (VEGF-A) is the most important modulator of angiogenesis^11,12^. Our previous reports have implicated the role of VEGF-A in the disease progression of chondrosarcoma^13^. Therefore, it is important to investigate the signaling cascades of VEGF-A production in human chondrosarcoma cells.

Resistin is a 12.5-kDa cysteine-rich adipokine that is constitutively secreted by adipose tissue^14^; resistin levels in plasma correlate with inflammatory markers and coronary artery calcification, a measure of coronary atherosclerosis^15^. Accumulating evidence indicates that resistin regulates tumor progression and metastasis^16^. It has been reported that resistin is a high-risk regulator for the development of renal cell carcinoma^17^, while in colorectal cancer, the levels of resistin in serum strongly correlates with tumor stage^18^. Our previous work indicates that resistin regulates metastasis in chondrosarcoma, enhancing chondrosarcoma cell migration by increasing levels of MMP-2 expression^19^. Resistin has also been found to enhance lymphatic endothelial cell-associated lymphangiogenesis in human chondrosarcoma in vitro and in vivo^20^. However, the role of resistin in tumor angiogenesis is largely unknown. In this study, we examined the relationship of resistin with VEGF-A expression and tumor angiogenesis, and further investigated the molecular mechanism underlying resistin-induced VEGF-A-dependent angiogenesis in chondrosarcoma microenvironment.

## Results

### Resistin promotes VEGF-A-dependent EPCs angiogenesis

**Fig. 1:**
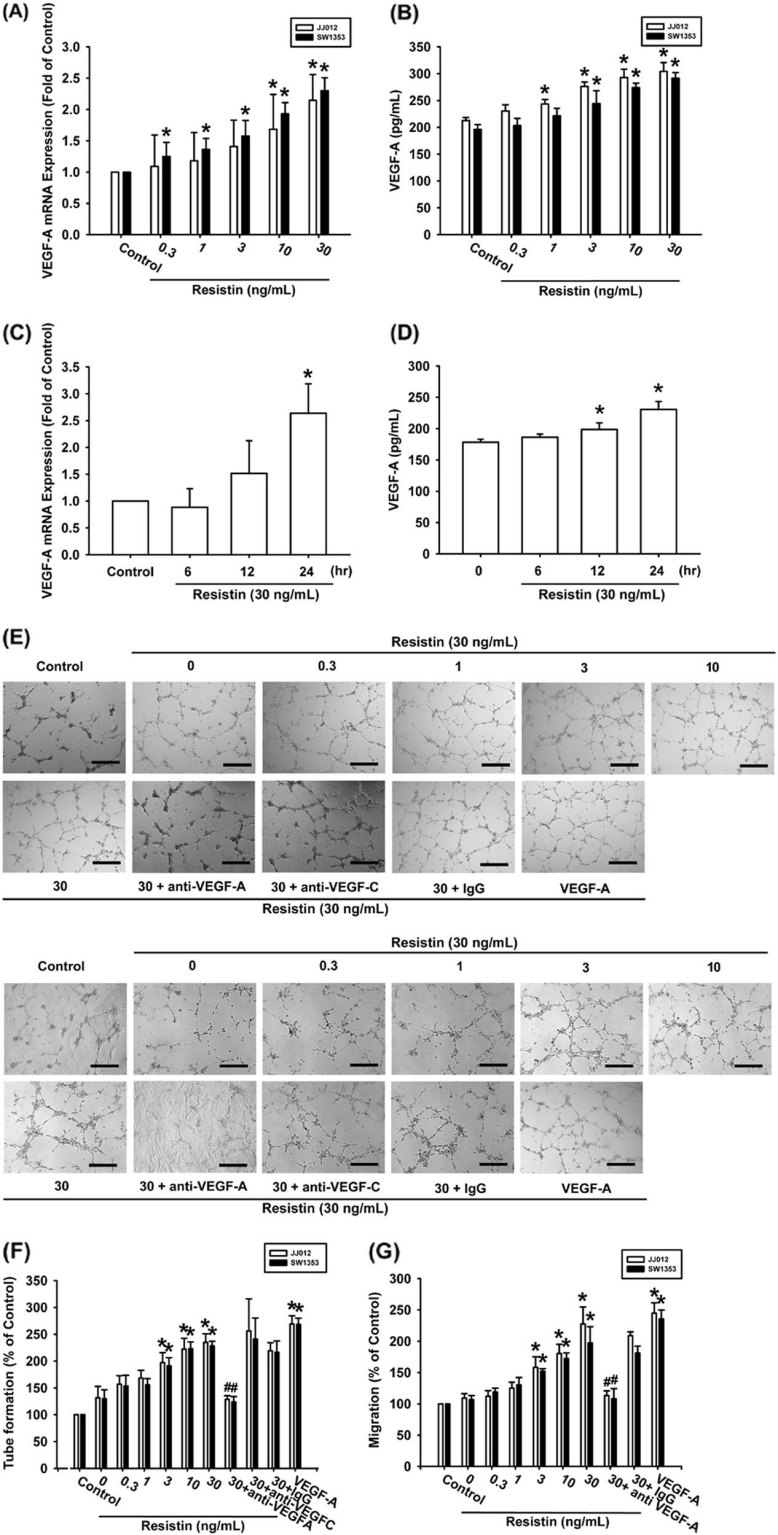
Resistin promotes VEGF-A production and angiogenesis in human chondrosarcoma. **a**–**d** Chondrosarcoma cells were incubated with resistin (0.3–30 ng/ml) for 24 h or stimulated with resistin (30 ng/ml) for indicated time intervals; VEGF-A expression was examined by qPCR and ELISA. **e**–**g** Chondrosarcoma cells were incubated with resistin for 24 h then stimulated with VEGF-A, VEGF-C, or IgG antibody (1 μg/ml) for 30 min. The conditioned medium (CM) was then collected and applied to endothelial progenitor cells (EPCs). EPCs tube formation and migration were measured (Size bar = 200 μm). Results are expressed as the mean ± SEM. *p < 0.05 as compared with the control group; #*p* < 0.05 as compared with the resistin-treated group

We have previously reported that resistin enhances tumor metastasis and lymphangiogenesis in human chondrosarcoma cells^19,20^. Here, we examined the roles of resistin in VEGF-A expression and the angiogenic process. Directly applying resistin to chondrosarcoma cell lines (JJ012 and SW1353) promoted mRNA and VEGF-A protein expression in a concentration-dependent manner (Fig. 1a, b), while stimulating chondrosarcoma cell lines with resistin (30 ng/ml) facilitated VEGF-A expression in a time-dependent manner (Fig. 1c, d). The effects of resistin-mediated angiogenesis in chondrosarcoma cells were evaluated by EPCs migration and tube formation assays^21^. CM from resistin-treated chondrosarcoma cells enhanced migration and tube formation in EPCs (Fig. 1e–g). Resistin-induced EPCs migration and tube formation was abolished by VEGF-A mAb, whereas VEGF-C mAb had no such effects (Fig. 1f, g), which suggests that resistin induces angiogenesis in a VEGF-A-dependent manner.

### The PI3K/Akt signaling pathway plays a role in resistin-induced VEGF-A expression

**Fig. 2:**
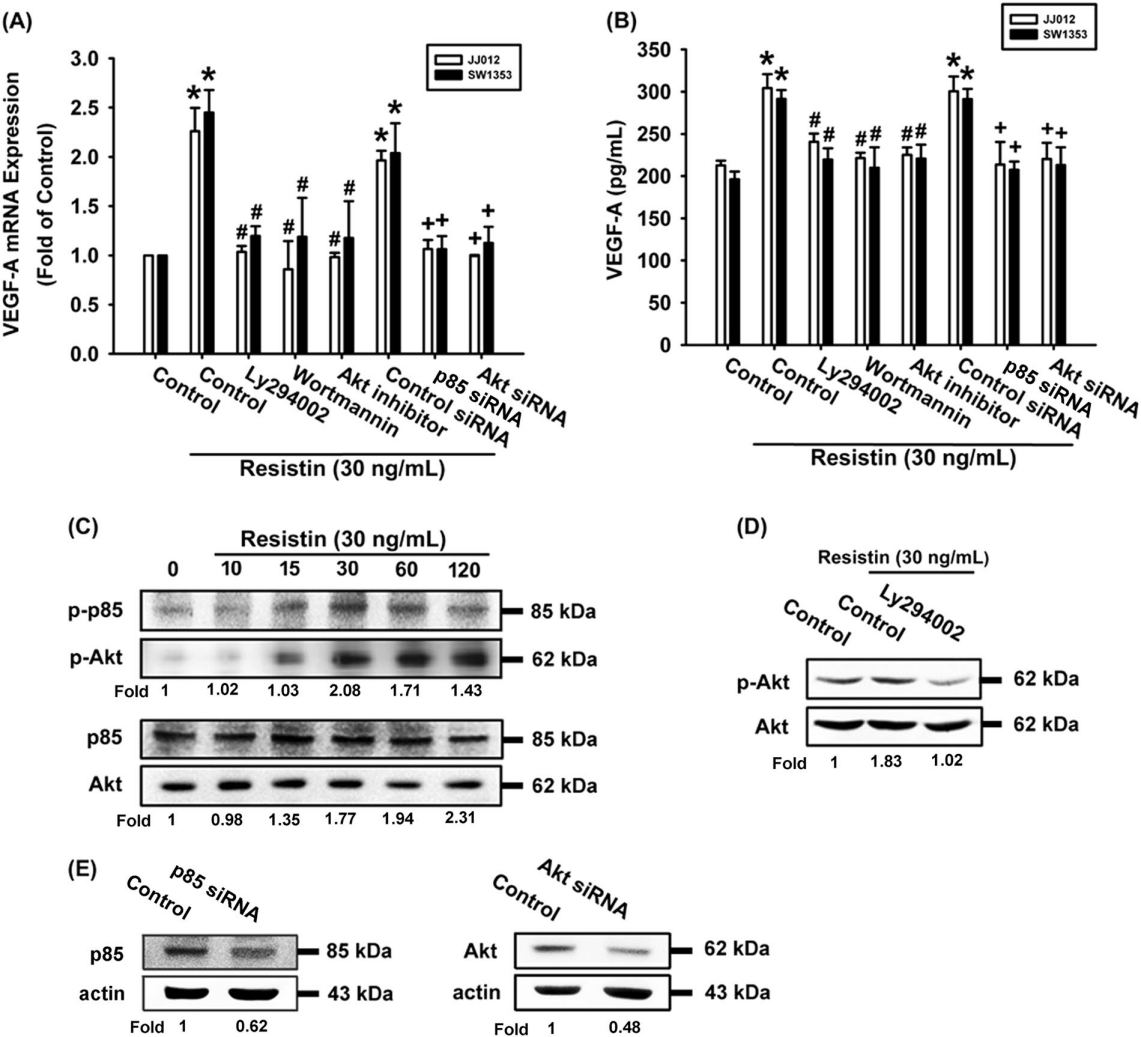
The PI3K/Akt pathway is involved in resistin-induced VEGF-A expression in human chondrosarcoma cells. **a**, **b** Cells were pretreated for 30 min with Ly294002 (10 μM), wortmannin (5 μM), and an Akt inhibitor (10 μM), or transfected with p85 or Akt siRNA then stimulated with resistin. The VEGF-A expression was examined by qPCR and ELISA. **c** JJ012 cells were incubated with resisitin, the p-p85 and Akt expression was examined by Western blot. **d** JJ012 cells were pretreated with Ly294002 for 30 min, then stimulated with resistin and Akt phosphorylation was examined. **e** JJ012 cells were transfected with p85 or Akt siRNA, the p85 and Akt expression was examined. Results are expressed as the mean ± SEM. *p < 0.05 as compared with the control group; #*p* < 0.05 as compared with the resistin-treated group; +p < 0.05 as compared with the control siRNA group

The PI3K/Akt signaling pathway is commonly implicated in the angiogenesis and metastasis of different tumor cells^13,22,23^. Pretreating chondrosarcoma cells with PI3K inhibitors (Ly294002, wortmannin) or an Akt inhibitor abolished resistin-enhanced VEGF-A expression (Fig. 2a, b). PI3K and Akt siRNA showed similar effects; transfection of cells with p85 or Akt siRNAs inhibited p85 and Akt expression (Fig. 2a–e). The PI3K-dependent signaling pathway is known to enzymatically activate Akt residue phosphorylation^24^. When we investigated p85 and Akt phosphorylation in response to resistin treatment, we identified a significant, time-dependent induction of p85 and Akt phosphorylation (Fig. 2c). Moreover, resistin-induced Akt phosphorylation was inhibited when cells were pretreated with a PI3K inhibitor (Fig. 2d). It appears that resistin acts via PI3K/Akt-dependent signaling pathway to increase the expression of VEGF-A in human chondrosarcoma cells.

### Resistin facilitates VEGF-A-related angiogenesis by suppressing miR-16-5p

**Fig. 3:**
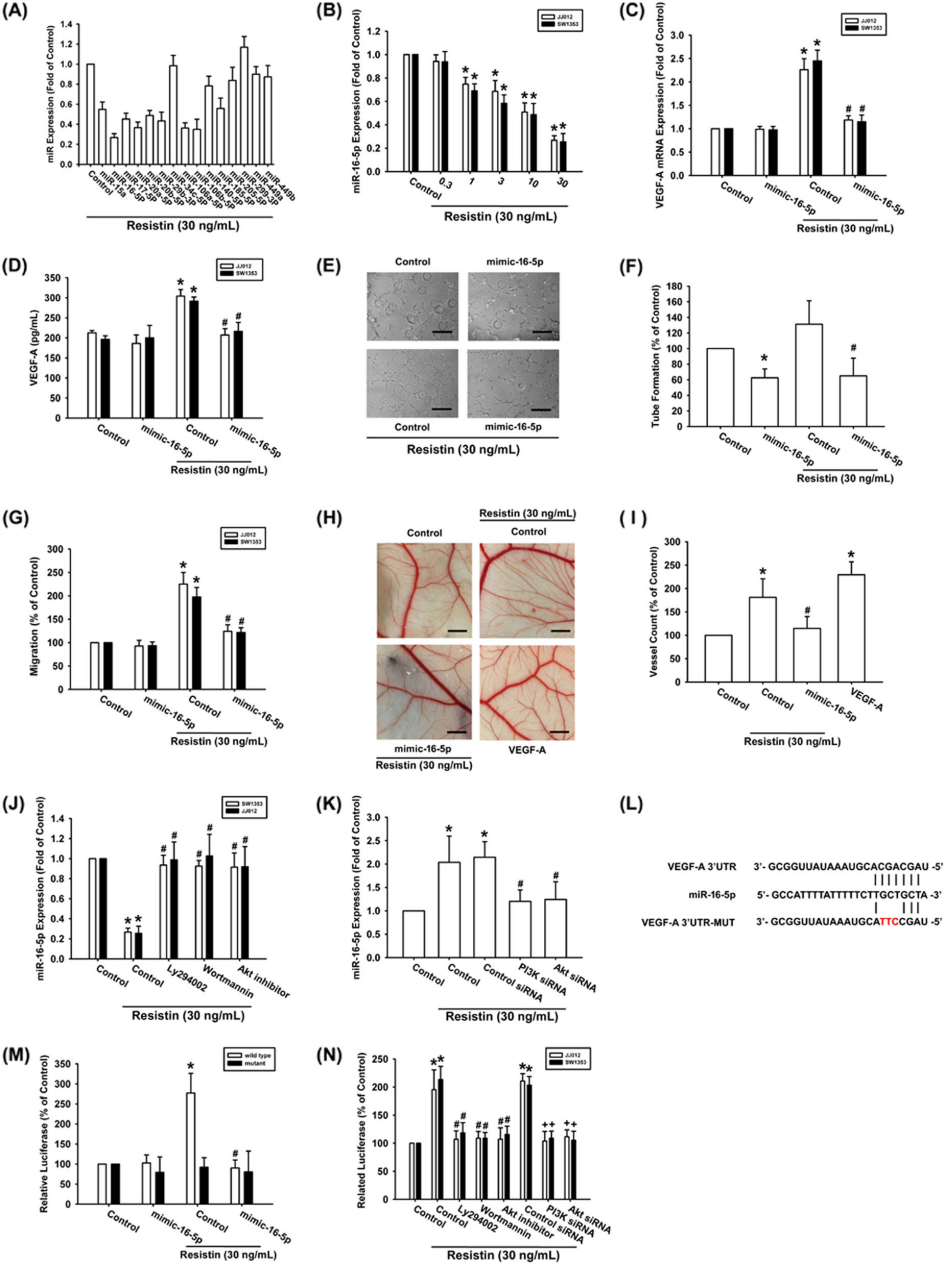
Resistin promotes VEGF-A and angiogenesis via inhibiting miR-16-5p. **a**, **b** Cells were incubated with resistin for 24 h and the indicated miRNAs expression was examined by qPCR. **c**, **d** Cells were transfected with the miR-16-5p mimic then incubated with resisin for 24 h; VEGF-A expression was measured by qPCR and ELISA. **e**–**g** The CM was applied to EPCs and analyzed for tube formation and migration activity (Size bar = 200 μm). **h**, **i** The CM also applied to chick embryos for 4 days and then resected, fixed, and photographed with a stereomicroscope (Size bar = 1 mm). **j**, **k** Cells were treated with PI3K and Akt inhibitors or siRNAs then incubated with resisin for 24 h; The miR-16-5p expression was measured by qPCR. **l** VEGF-A-3′UTR luciferase plasmids contain the miR-16-5p binding site. **m**, **n** Cells were treated with PI3K and Akt inhibitors or siRNAs then incubated with resisin for 24 h; The VEGF-A luciferase activity was measured. Results are expressed as the mean ± SEM. *p < 0.05 as compared with the control group; #*p* < 0.05 as compared with the resistin-treated group; +p < 0.05 as compared with the control siRNA group

Accumulated evidences suggest that miRNAs are the crucial regulator of VEGF-A production and EPCs angiogenesis^25,26^. Use of open-source software in this study to predict and identify target miRNAs found that the 3′UTR region of VEGF-A mRNA harbors potential binding sites for 15 candidate miRNAs, and that miR-16-5p is markedly downregulated after resistin treatment (Fig. 3a). Exogenous resistin concentration-dependently inhibited miR-16-5p expression (Fig. 3b). Transfection of cells with miR-16-5p mimic diminished resistin-enhanced VEGF-A expression (Fig. 3c, d) and also inhibited resistin-boosted EPCs migration and tube formation (Fig. 3e–g). When resistin-regulated angiogenesis was examined by the in vivo CAM assay, we observed that CM from the resistin-treated chondrosarcoma cells promoted vessel formation, which was diminished by miR-16-5p mimic (Fig. 3h, i).

Next, we examined the relationship between PI3K/Akt pathway and miR-16-5p. Treatment of cells with PI3K and Akt inhibitors or siRNA reversed the resistin-induced reduction in miR-16-5p expression (Fig. 3j, k). We further determined whether miR-16-5p governs the 3′UTR region of VEGF-A (Fig. 3l). The data showed that resistin-enhanced wile-type but not mutant VEGF-A-3′UTR luciferase activity (Fig. 3m). Incubation with PI3K and Akt inhibitors or siRNAs reversed resistin-mediated VEGFA-3′UTR luciferase activity (Fig. 3n), indicating that miR-16-5p impedes VEGF-A production via binding to 3′UTR region of the human *VEGF-A* gene through PI3K/Akt signaling pathway.

### Overexpression of resistin promotes VEGF-A-associated tumor angiogenesis

**Fig. 4:**
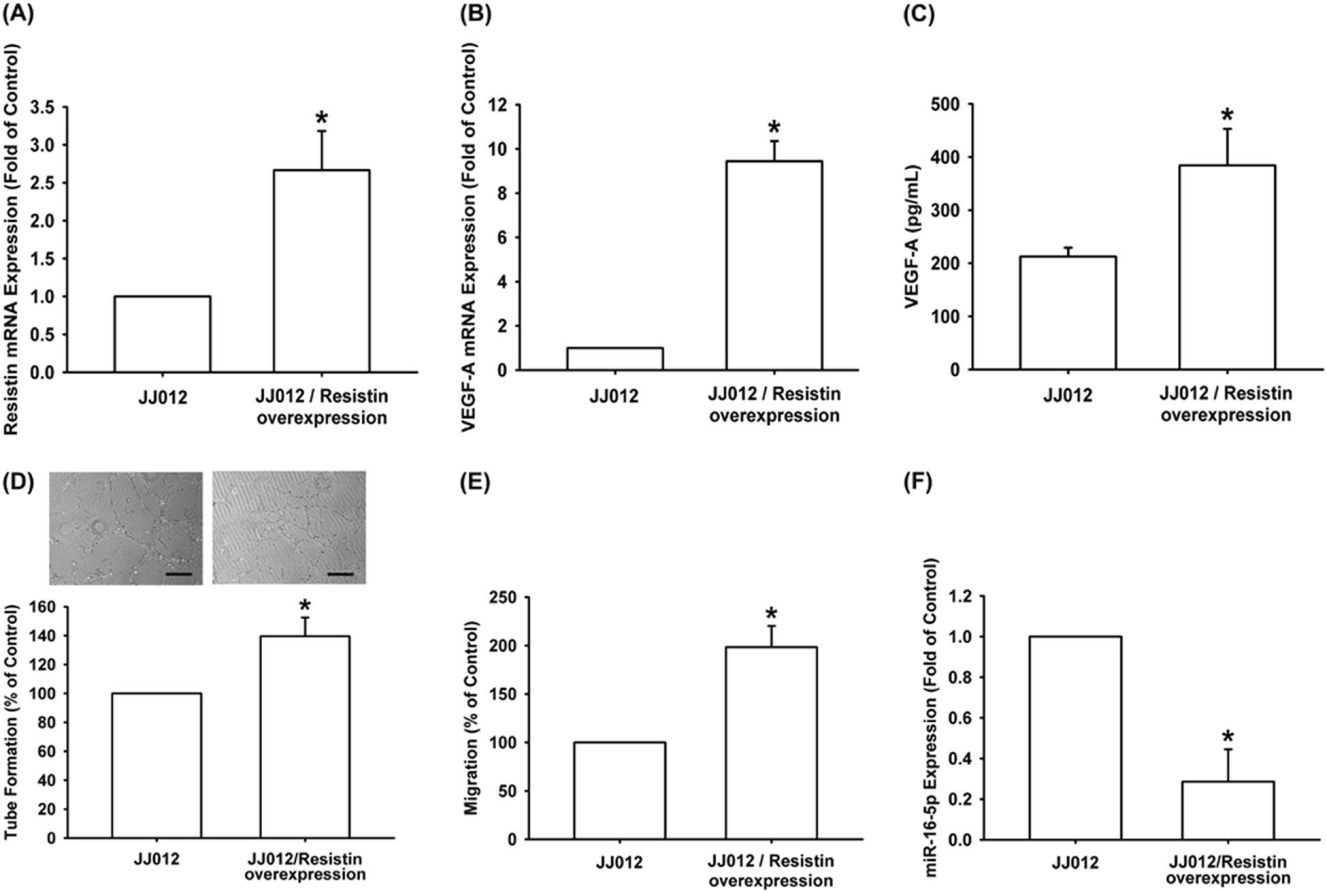
Overexpression of resistin promotes VEGF-A expression and angiogenesis. **a**–**c** Resistin and VEGF-A expression was examined by qPCR and ELISA in the indicated cells. **d**, **e** The CM was applied to EPCs and analyzed for tube formation and migration activity (Size bar = 200 μm). **f** miR-16-5p expression was examined by qPCR in the indicated cells. Results are expressed as the mean ± SEM. *p < 0.05 as compared with the control group; #*p* < 0.05 as compared with the resistin-treated group

**Fig. 5:**
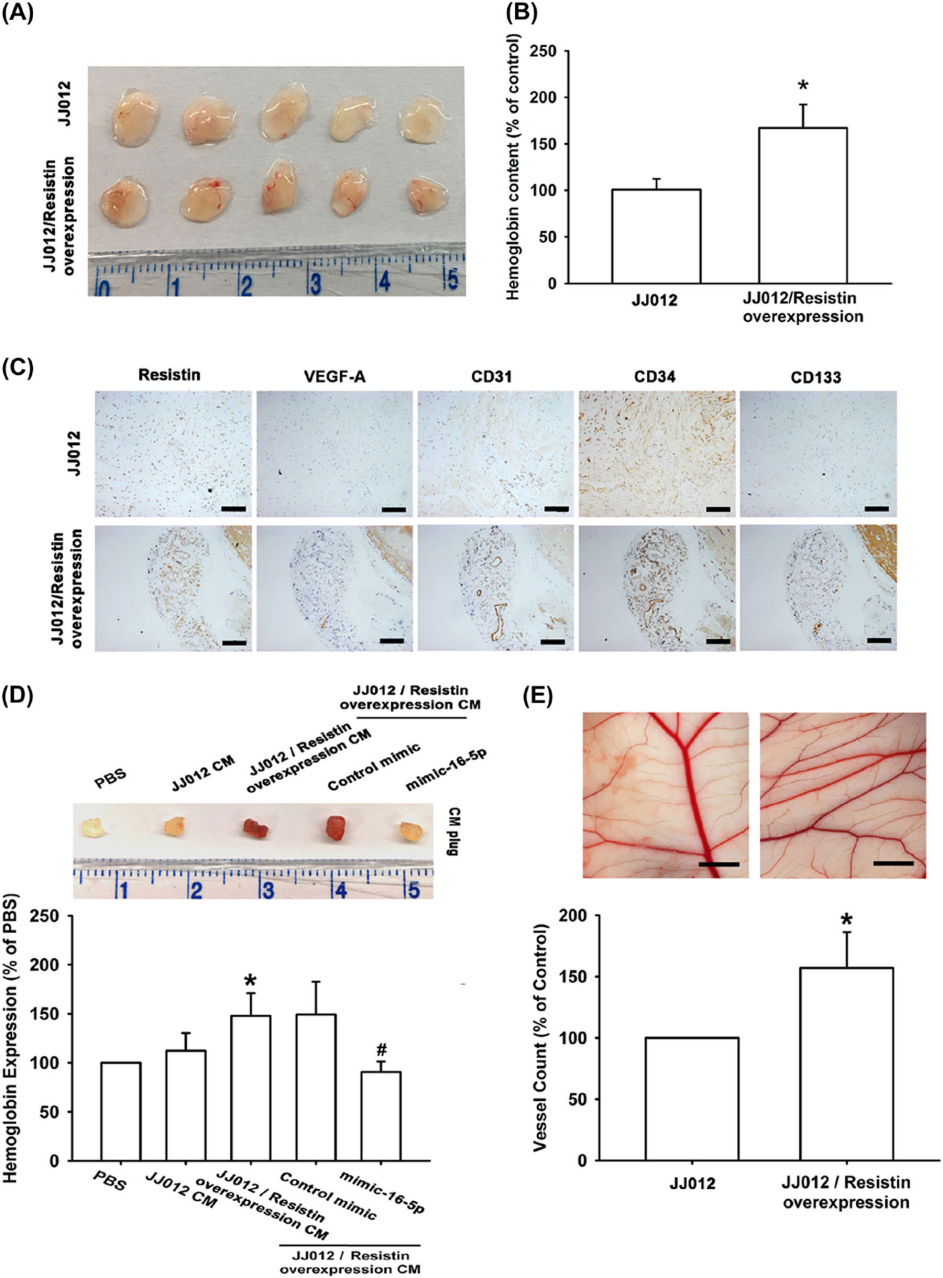
Overexpression of resistin induces VEGF-A-dependent angiogenesis by inhibiting miR-16-5p in vivo. **a**, **b** Mice (*n* = 10 each group) were injected subcutaneously with the indicated cells. At 14 days after injection, the tumors were excised, photographed with a microscope, and hemoglobin levels were measured. **c** Tumor sections were immunostained with resistin, VEGF-A, CD31, CD34, and CD133 antibodies (Size bar = 100 μm) **d** Matrigel plugs were treated with indicated CM and subcutaneously injected into the flanks of nude mice. After 7 days, the plugs were photographed and hemoglobin levels were examined. **e** The indicated CM was applied to chick embryos for 4 days then resected, fixed, and photographed with a stereomicroscope (Size bar = 1 mm) (*n* = 7). Results are expressed as the mean ± SEM. *p < 0.05 as compared with the control group; #*p* < 0.05 as compared with the resistin-treated group

To confirm the resistin-induced promotion of VEGF-A expression and angiogenesis in vivo, resistin-overexpressing JJ012 cells were established^20^. Overexpression of resistin enhanced the expression of resistin and VEGF-A (Fig. 4a–c), while CM collected from resistin-overexpressing JJ012 cells facilitated EPCs migration and tube formation (Fig. 4d, e). Conversely, miR-16-5p expression was diminished by resistin-overexpressing human chondrosarcoma cells (Fig. 4f). Next, we examined whether overexpression of resistin-promoted tumor-associated angiogenesis in vivo. Analysis of the tumor hemoglobin content revealed that resistin overexpression promoted chondrosarcoma-induced angiogenesis in vivo (Fig. 5a, b). Immunohistochemical (IHC) staining revealed that resistin overexpression increased the expression of resistin, vessel markers VEGF-A and CD31, as well as EPC markers CD34 and CD133 (Fig. 5c). Resistin overexpression also enhanced vessel formation in vivo, according to Matrigel plug and CAM assay results (Fig. 5d, e).

### Correction of resistin, VEGF-A, and miR-16-5p in chondrosarcoma patients

**Fig. 6:**
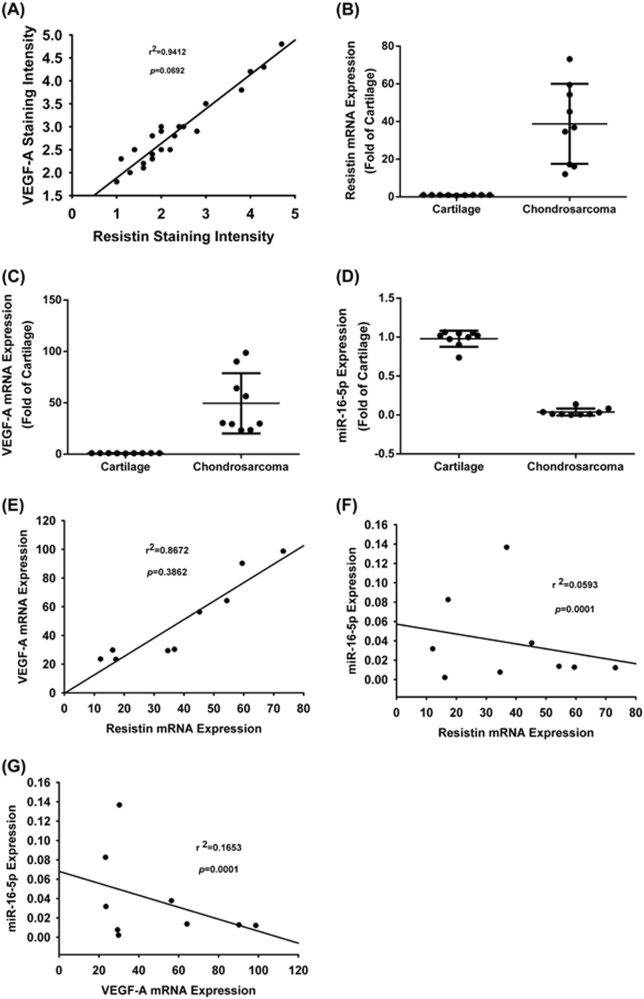
Correlations between resistin, VEGF-A, and miR-16-5p in chondrosarcoma patients. **a** Correlations between resistin and VEGF-A expression, and IHC data relating to resistin and VEGF-A expression, in normal cartilage and human chondrosarcoma tissue. **b**–**d** Levels of resistin, VEGF-A, and miR-16-5p mRNA expression was examined by qPCR in normal cartilage (*n* = 9) and chondrosarcoma tissue (*n* = 9). **e**–**g** Correlations between VEGF-A, resistin, and miR-16-5p in normal cartilage and chondrosarcoma tissue. Results are expressed as the mean ± SEM

Next, we evaluated resistin and VEGF-A expression in clinical chondrosarcoma samples. Resistin patterns correlated positively with VEGF-A in IHC-stained chondrosarcoma specimens (Fig. 6a). qPCR analysis indicated higher levels of resistin and VEGF-A expression in tumor specimens compared with normal cartilage (Fig. 6b, c) and lower levels of miR-16-5p expression in tumor specimens compared with normal tissue (Fig. 6d). The expression of resistin was significantly correlated with VEGF-A levels, while the content of miR-16-5p was negatively correlated with the expression of resistin and VEGF-A in human chondrosarcoma specimens (Fig. 6e–g). Our results demonstrate that resistin promotes VEGF-A expression by suppressing miR-16-5p in chondrosarcoma patients.

## Discussion

**Fig. 7:**
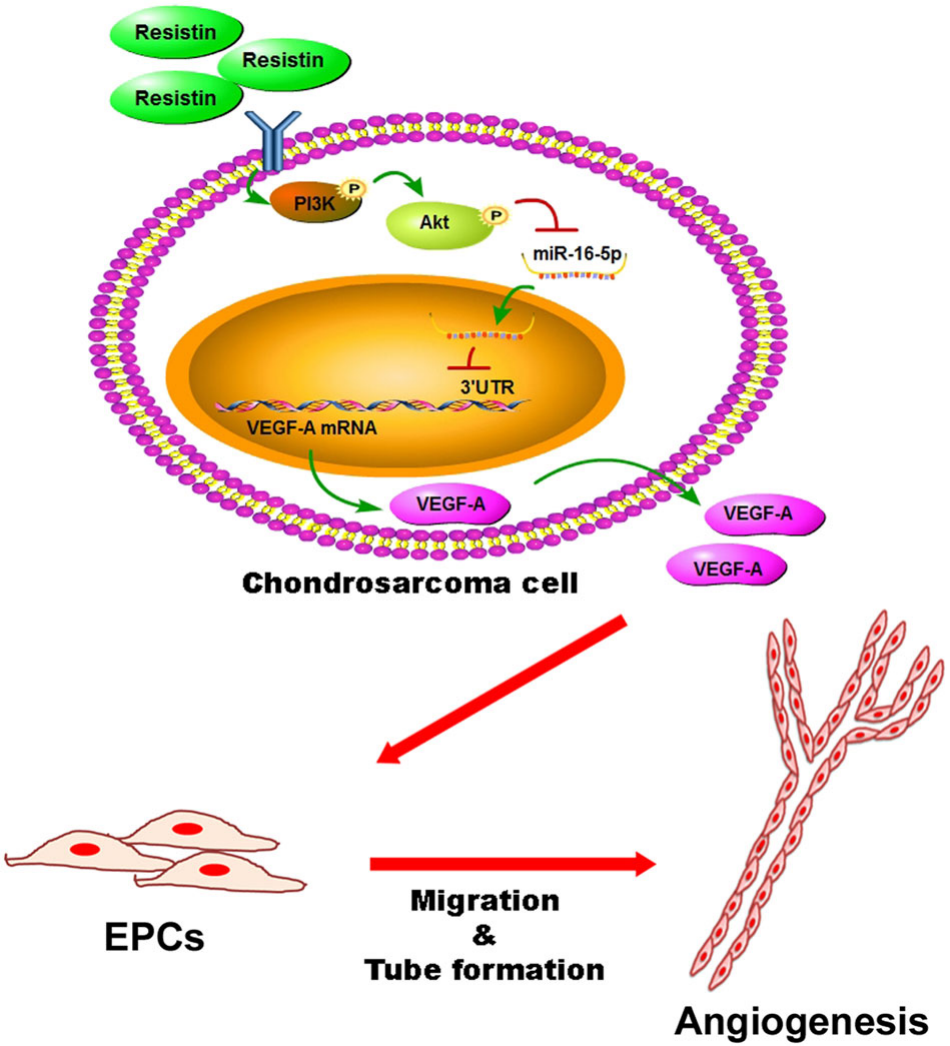
Schematic diagram summarizes the mechanism of resistin-boosted tumor angiogenesis in chondrosarcoma microenvironment. Resistin promotes VEGF-A production by downregulating miR-16-5p through PI3K/Akt-dependent pathway in human chondrosarcoma cells, and subsequently induces EPCs angiogenesis

Chondrosarcomas are a group of heterogeneous, malignant bone neoplasms that constitute around 26% of all bone cancers^27,28^. Metastatic propensity of human chondrosarcomas highly correlates with the pathological tumor stages. Surgery is the favored therapeutic option for chondrosarcoma; chemotherapy and radiotherapy have very limited effectiveness^29^. Many low- and moderate-grade chondrosarcomas have a relatively indolent growth rate; ~15% of all metastasis-caused deaths occur more than 5 years after first diagnosis^30^. This characteristic offers an important opportunity for an effective adjuvant therapy to prevent metastatic disease in chondrosarcoma. We have previously reported that resistin enhances tumor metastasis and lymphangiogenesis in human chondrosarcoma cells^19,20^. We hypothesized that resistin would influence tumor angiogenesis in chondrosarcoma microenvironment. In this study, we provide evidences that resistin induces VEGF-A production in human chondrosarcoma cells, and contributes to tumor angiogenesis by suppressing miR-16-5p expression through PI3K/Akt signaling pathway (Fig. 7). This is the first indication that adipokine resistin boosts VEGF-A-associated tumor angiogenesis via downregulation of miR-16-5p in vitro and in vivo.

Resistin is an adipokine that is associated with obesity, inflammation, and various cancers^19,21,31^. In patients with lung cancer, high serum resistin levels may play a role in the pathogenesis of cancer cachexia^32^. Upregulation of resistin in serum has been detected in oral cancer patients^33^ and resistin overexpression or upregulation has been observed in various human cancers, such as renal cell carcinoma, chondrosarcoma, and colon cancer^17,18,20^. In addition, resistin plays a critical role in breast cancer progression, drug resistance, and metastasis^34–36^. In this study, our results suggest that higher levels of resistin expression are found in chondrosarcoma tissue than in normal cartilage. In addition, resistin expression was positively correlated with VEGF-A levels. We have previously reported that inhibition of resistin reduces chondrosarcoma metastasis and lymphangiogenesis^19,20^. These combined results suggest that inhibition of resistin might be a valuable therapeutic strategy for chondrosarcoma.

Activation of the PI3K/Akt signaling pathway is the critical event in many types of cancer and represents a potential therapeutic target against cancer growth. This pathway mediates multiple cellular functions, including cell survival, proliferation, migration, and autophagy^37^. Furthermore, the PI3K/Akt signaling pathway is associated with tumor angiogenesis. For instance, adiponectin promotes VEGF-A-dependent angiogenesis in chondrosarcoma through the PI3K/Akt cascade^13^. In this study, we demonstrated that PI3K and Akt inhibitors are capable of inhibiting resistin-induced VEGF-A expression. Furthermore, we observed that p85 and Akt siRNAs reduced VEGF-A expression in chondrosarcoma cells. When we incubated cells with resistin, we found an increase in the phosphorylation of PI3K and Akt. Pretreatment of cells with a PI3K inhibitor repressed resistin-induced Akt phosphorylation. This indicates that the PI3K/Akt signaling pathway is involved in resistin-mediated VEGF-A expression and angiogenesis. MAPK and HIF-1α signaling are major pathways involved in angiogenesis process^38,39^. MAPK and HIF-1 inhibitors all abolished resistin-induced VEGF-A expression and EPCs tube formation (Supplementary data Fig. S1), indicating MAPK and HIF-1α also mediated resistin-promoted angiogenesis.

Patients with metastatic chondrosarcoma have a very poor prognosis, so it is important to find a means of preventing metastasis^19^. Our results highlight new insights into resistin functions in the angiogenic and metastatic process. Upregulation of resistin expression promotes EPCs angiogenesis in chondrosarcoma. The mechanisms involved in resistin-induced angiogenesis remain unclear, although our findings show that resistin downregulates miR-16-5p via the PI3K/Akt signaling pathway. This study emphasizes the importance of resistin in chondrosarcoma angiogenesis and suggests that resistin may be a useful target in the management of chondrosarcoma.

## Materials and methods

### Materials

The recombinant human resistin was purchased from R&D Systems (Minneapolis, MN, USA). We purchased p85, Akt, and β-actin primary antibodies (Santa Cruz Biotechnology, CA, USA), as well as rabbit polyclonal antibodies specific for p-p85 and p-Akt (Cell Signaling Technology, Danvers, MA, USA). The miR-16-5p mimic, miRNA control, Lipofectamine 2000, and Trizol were purchased from Life Technologies (Carlsbad, CA, USA). Dharmacon Research (Lafayette, CO, USA) supplied ON-TARGETplus siRNAs. Gibco-BRL life technologies (Grand Island, NY, USA) supplied fetal bovine serum (FBS), DMEM, α-MEM, and all other cell culture reagents. Promega (Madison, WI, USA) supplied the pSV-β-galactosidase vector and luciferase assay kits. All other chemicals or inhibitors were purchased from Sigma-Aldrich (St. Louis, MO, USA).

### Cell culture

The human chondrosarcoma cell line (JJ012) was kindly supplied by Dr. Sean P. Scully’s laboratory at the University of Miami School of Medicine (Miami, FL, USA). SW1353 human chondrosarcoma cell line was purchased from the American Type Culture Collection (Manassas, VA, USA). Chondrosarcoma cell culture conditions were recorded as previously described^40^. Human EPCs were isolated and cultured by a standard method as previously described^41,42^. This study was approved by the Institutional Review Board of Mackay Medical College, New Taipei City, Taiwan (P1000002).

### Preparation of conditioned medium (CM) and ELISA assay

Human chondrosarcoma cells were treated with resistin alone for 24 h, or pretreated with pharmacological inhibitors or transfected with siRNA followed by stimulation with resistin for 24 h. After treatment, the cells were washed and changed to serum-free medium. CM was then collected 2 days after the change of medium and stored at −80 °C until use. The production of VEGF-A was determined by VEGF-A ELISA kit, according to the procedure described by the manufacturer.

### EPCs tube formation assay

The capillary tube formation assay was carried out on Matrigel-coated (BD Biosciences, Bedford, MA, USA) 48-well plates. Measurement of tube formation was performed to examine the differentiation and formation of capillary-like tubules on EPCs according to previously described procedures^40^.

### EPCs migration assay

Transwell inserts (8-μm pore size; Costar, NY, USA) were used for migration determination. EPCs migratory ability was assayed by the method based on our previous work^40^.

### Western blot analysis

Cell lysates underwent electrophoresis with SDS-PAGE and were transferred to PVDF membranes according to the method described in our previous studies^43,44^. After blocking the blots with 4% bovine serum albumin, the blots were treated with primary antibody and then peroxidase-conjugated secondary antibody consecutively. Visualizations of the blots were accomplished by enhanced chemiluminescence with UVP Biospectrum system (UVP, Upland, CA, USA).

### Quantitative real-time PCR (qPCR) of mRNA and miRNA

Total RNA was extracted from chondrosarcoma cells using TRIzol reagent. The qPCR analysis was carried out according to an established protocol^20^.

### Chick chorioallantoic membrane (CAM) assay

Fertilized chicken eggs were used in CAM assay. In vivo angiogenesis was determined by a standard method as described previously^42^.

### Matrigel plug assay

Four-week-old male nude mice (National Laboratory Animal Center, Taipei, Taiwan) were subcutaneously injected with 0.15 ml of Matrigel containing the indicated chondrosarcoma CM. On day 7, the Matrigel plugs were harvested and the hemoglobin concentrations were evaluated using Drabkin’s method (Drabkin’s Reagent Kit, Sigma–Aldrich).

### Plasmid construct and reporter assay

We obtained wild-type (WT)-VEGFA-3′-UTR and mutant-type (MUT)-VEGFA-3′-UTR DNA fragments from Invitrogen (Carlsbad, CA, USA) and subcloned into the pmirGLO-control luciferase reporter vector (Promega). Luciferase activity was assayed by the method based on our previous work^27^.

### In vivo tumor xenograft model

Nude mice (4-week of age) were purchased from the National Laboratory Animal Center. All animal experiments were done in accordance with a protocol approved by China Medical University’s Institutional Animal Care and Use Committee (IACUC Approval No. 104-154-N). Normal or resistin-overexpressing JJ012 cells harvested from exponentially growing cell cultures were implanted into the right flanks of mice by subcutaneous injection of 2 × 10^6^ cells resuspended in 200 μL of 50% serum-free medium and 50% Matrigel. After 14 days, the tumors were removed and fixed in 10% formalin.

### Immunohistochemical (IHC) staining

Tumor samples were deparaffinized with xylene and rehydrated with ethanol. IHC analysis was performed to detect the expression of resistin and angiogenic markers according our previous protocol^20^.

### Patients and specimen preparations

Human cartilage specimens were obtained during primary total knee arthroplasty. Tumor specimens were collected from patients diagnosed with chondrosarcoma who underwent orthopedic surgery at China Medical University Hospital. Normal cartilage and chondrosarcoma tissues were used in IHC and q-PCR assays. All study participants gave written consent before enrollment. The study protocol was approved by China Medical University Hospital’s Institutional Review Board (CMUH103-REC2-023, CMUH 104-REC2-055).

### Statistical analysis

Data are presented as mean ± standard error of the mean (SEM) of at least three independent experiments. The Student’s *t*-test determined statistical differences between samples and the Bonferroni post hoc procedure was performed for a one-way analysis of variance (ANOVA) of statistical comparisons between more than two samples, and *p*-values less than 0.05 were considered significant.

## Supplementary information

Supplementary Data
