# Supplementary material for: Resistin facilitates VEGF-A-dependent angiogenesis by inhibiting miR-16-5p in human chondrosarcoma cells
Source: Cell Death Dis. 2019 Jan 10;10(1):31. doi: 10.1038/s41419-018-1241-2 (PMC6328541; doi:10.1038/s41419-018-1241-2)
Supplement: Supplementary file 1 — Supplementary Data [file 41419_2018_1241_MOESM1_ESM.doc]

**Supplementary** **Data**


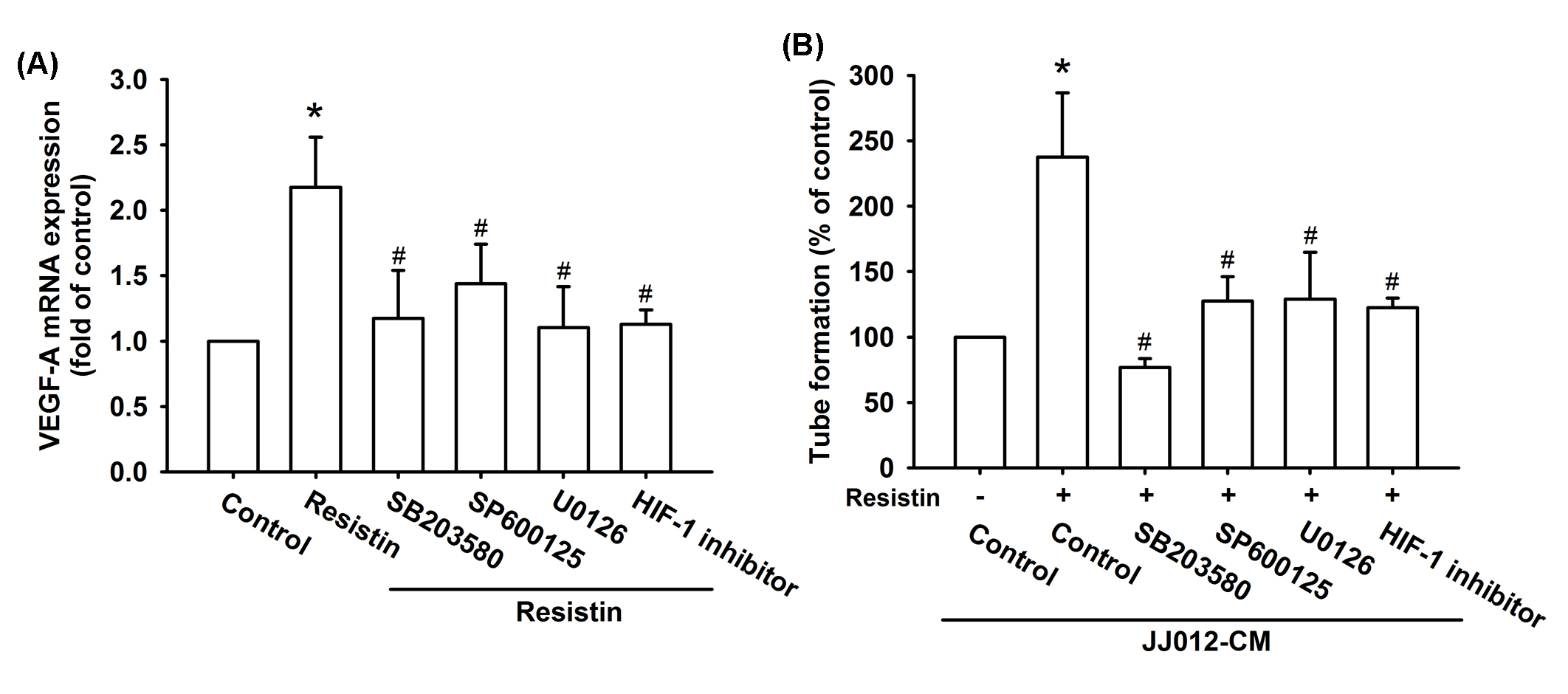


**Figure S1. MAPK and HIF-1α were involved in resistin-promoted angiogenesis.** (A) JJ012 cells pretreated for 30 min with p38 (SB203580), JNK (SP600125), ERK (U0126) and HIF-1 inhibitor then stimulated with resistin (30 ng/ml). The VEGF-A expression was examined by qPCR. (B) The CM was applied to EPCs and analyzed for tube formation. Results are expressed as the mean ± SEM. **p* < 0.05 as compared with the control group; #*p* < 0.05 as compared with the resistin-treated group.
